# PySupercharge: a python algorithm for enabling ABC transporter bacterial secretion of all proteins through amino acid mutation

**DOI:** 10.1186/s12934-024-02342-z

**Published:** 2024-04-20

**Authors:** Yerin Kim, Danny Kim, Nguyen-Mihn Hieu, Hyunjong Byun, Jung Hoon Ahn

**Affiliations:** 1 Department of Chemistry and Biology, Korea Science Academy of Korea Advanced Institute of Science and Technology, Busan, South Korea; 2 Department of Biological Sciences, Korea Advanced Institute of Science and Technology (KAIST), Daejeon, South Korea

**Keywords:** Python Algorithm, Supercharging, Protein production, Secretion, ABC Transporter

## Abstract

**Background:**

The process of producing proteins in bacterial systems and secreting them through ATP-binding cassette (ABC) transporters is an area that has been actively researched and used due to its high protein production capacity and efficiency. However, some proteins are unable to pass through the ABC transporter after synthesis, a phenomenon we previously determined to be caused by an excessive positive charge in certain regions of their amino acid sequence. If such an excessive charge is removed, the secretion of any protein through ABC transporters becomes possible.

**Results:**

In this study, we introduce ‘linear charge density’ as the criteria for possibility of protein secretion through ABC transporters and confirm that this criterion can be applied to various non-secretable proteins, such as SARS-CoV-2 spike proteins, botulinum toxin light chain, and human growth factors. Additionally, we develop a new algorithm, PySupercharge, that enables the secretion of proteins containing regions with high linear charge density. It selectively converts positively charged amino acids into negatively charged or neutral amino acids after linear charge density analysis to enable protein secretion through ABC transporters.

**Conclusions:**

PySupercharge, which also minimizes functional/structural stability loss of the pre-mutation proteins through the use of sequence conservation data, is currently being operated on an accessible web server. We verified the efficacy of PySupercharge-driven protein supercharging by secreting various previously non-secretable proteins commonly used in research, and so suggest this tool for use in future research requiring effective protein production.

**Supplementary Information:**

The online version contains supplementary material available at 10.1186/s12934-024-02342-z.

## Background

Reengineering protein amino acid sequences to decrease electric charge, or “supercharging”, can improve secretion ability of the protein through the bacterial ABC transporter system, which is an improved alternative to previous protein production methods [1–3]. Here, we introduce PySupercharge, an automated Python algorithm that supercharges protein sequences to be compatible for ABC transporter secretion.

The conventional method for protein production widely used in academia and industry utilizes non-secretory *Escherichia coli* systems [4, 5]. In this process, *E. coli* is transformed with the target protein’s expression vector, and the target protein is extracted through cell lysis. This *E. coli* system is widely used for its production fidelity [6, 7]. However, contaminant proteins are inevitably added during the lysis process, making multiple steps such as gel filtration and column chromatography necessary. This disadvantage of *E. coli* protein production systems is yet to be resolved. One of the alternative methods used is the secretion of proteins into the extracellular space [8, 9]. This secretion method allows for a simpler purification process and guarantees the production of proteins in their correctly folded, active forms [10].

Many gram-negative bacteria including *E. coli* and *Pseudomonas fluorescens* have the Type I secretion system (T1SS), [11, 12]. T1SS is composed of three proteins: an ATP-binding cassette (ABC) protein, a membrane fusion protein (MFP), and an outer membrane protein (OMP) [13]. Unfolded protein is secreted into the extracellular space, where it folds back to its original form [14]. A broad range of heterogenous proteins can be secreted by T1SS, such as various growth factors, green fluorescent protein (GFP), and enzymes [2, 15–19]. Since the T1SS secretes the target protein to extracellular space and gram-negative bacteria do not secrete native proteins [20], this system is suitable for extracellular protein production. However, the bacterial secretion system including the T1SS is protein-dependent [21, 22]. Even if a secretion signal or a peptide secretion tag required by the secretion system is present, the secretion efficiency has been found to vary greatly by protein. This limitation would easily be resolved by finding the criteria for secretion, but it is still elusive. Various criteria have been studied, such as folding kinetics, the presence of an A/U rich sequence, the electric charge, and the isoelectric point [1, 23, 24]. Among these conditions, our previous research has focused on manipulating proteins’ electric charge.

In our previous examination of various secretable and non-secretable heterogenous proteins, it was found that non-secretable proteins almost always have high-density ‘excessively cationic regions’ [25]. We suggested that these regions have electrostatic limitations in being transported from the negatively charged intracellular space to the positively charged extracellular space. It was also shown that the artificial addition of excessively cationic regions to secretable proteins greatly reduces secretion efficiency. Therefore, determining whether the target protein has an excessively cationic region and neutralizing it can be critical for efficient protein production using bacterial secretion systems. Here, we introduce an automated algorithm, PySupercharge, for this process.

## Methods

### Linear charge density analysis

Unlike other supercharging tools that focus on the overall charge of the target protein, our method focuses on certain segments of a polypeptide sequence since proteins exit through T1SS in unfolded polypeptide form. Therefore, we utilize the local charge densities of the linearized amino acid sequence, which we called ‘linear charge density (LCD)’ in our previous study [25]. It is essentially the average charge of amino acids in a certain range or ‘window’ [25]. To express the criteria for excessively cationic regions more clearly, we used the total charge in a window instead of the average in this study. This modified LCD value of a window starting at initial position *i* is defined as *λ*_*i*_ by the following formula

$${\lambda }_{i} = \sum _{j=i}^{i+w-1}{q}_{j}$$

where *w* is the window width and *q*_*j*_ is the charge of the side chain of residue *j* at pH 7.

**Fig. 1:**
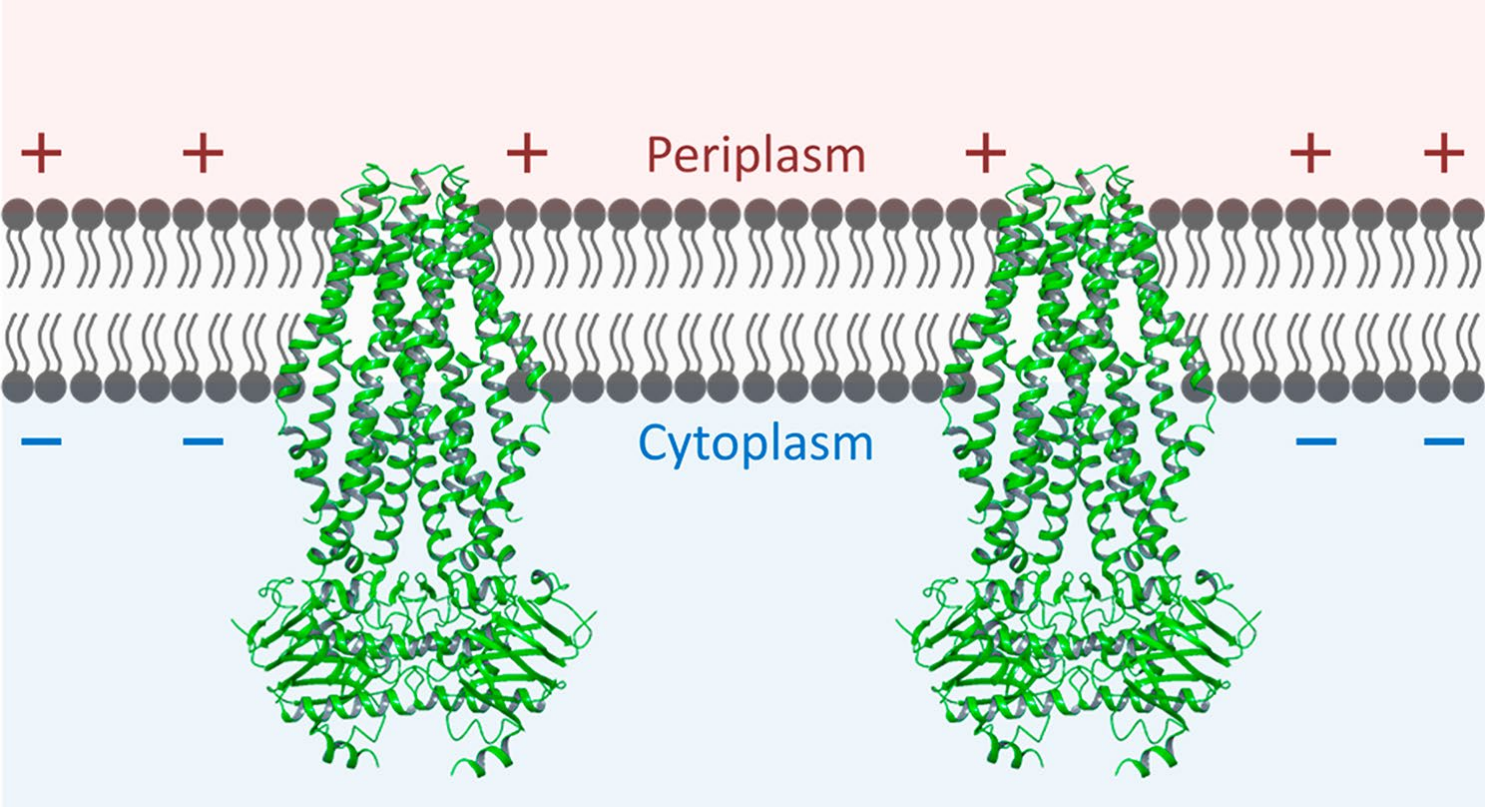
ABC Transporter Proteins. Structure of the ABC transporter protein in the bacterial inner membrane. The extracellular space (and by extension, the periplasmic space) is less negative than the cytoplasm [26]. This ABC transporter can be used in extracellular secretion-based protein production

From our previous study [25], we have determined that a window size of 20 amino acids is suitable for the *P. fluorescens* secretion system. This value was empirically determined based on the fact that 20 amino acids are influenced at a time by the membrane potential during transportation by the ABC transporter (Fig. 1). Also, the optimal LCD value cutoff for non-cationic-supercharged sequences was determined experimentally as ≤ (+ 2) in the window size of 20 amino acids.

The logic process of the main PySupercharge algorithm is simple. The amino acid sequence is analyzed, and the LCD value of a 20-amino-acid window is calculated for each available initial position $i$ in the sequence. Neutral amino acids are assigned zero charge, while positive amino acids (Lys, Arg) are assigned + 1 and negative amino acids (Glu, Asp) are assigned − 1. Histidine charge can be optionally configured to have a user-defined positive charge, as in some cases the small charge assignment might determine an excessively cationic sequence.

From here, the algorithm previously simply converted positive Lys or Arg to a random choice between Glu and Asp if a window’s LCD value exceeded the cutoff (there is not yet any concrete evidence that shows significant differences between Glu and Asp mutations. Cationic-to-anionic mutation was chosen for efficiency of supercharging). When the process was completed for every window, the cationic supercharged region would have been removed, resulting in a T1SS-secretable amino acid sequence.

However, this simple process of mutating amino acids based on LCD results had a limitation: changing an amino acid sequence by only using charge density information could cause losses in protein structure or function. Therefore, the effect of an amino acid mutation on a protein’s stability and functionality had to be calculated for consideration, which could be done by AvNAPSA (average number of neighboring atoms per side chain atom) and Consurf, respectively.

The tool we developed for linear charge density analysis is accessible on our webserver: https://mb.re.kr/apps/LCD.

### AvNAPSA implementation

A supercharging method, AvNAPSA (Liu et al., 2007), mutates flexible polar residues (DERKNQ) with the fewest average neighboring atoms per side chain atom [27]. We rewrote the original Perl script from the 2007 study in Python. Since AvNAPSA scores signify whether the amino acid residue has less interaction with other residues and is on the surface of the protein (a lower AvNAPSA score means fewer neighboring atoms and therefore less interaction), we utilized the AvNAPSA score to determine the effect on protein stability upon mutation. The positive-charge amino acids determined by LCD analysis were now only mutated if they had an AvNAPSA score in a certain range. An AvNAPSA cutoff of < 150 has been widely used in heavy supercharging to maintain protein stability while also allowing enough mutations to occur (a more stringent cutoff of < 100 is commonly used in moderate supercharging) [28].

### Consurf implementation

Consurf is a tool that traces the evolutionary history of amino acids in polypeptides and identifies conserved regions that are important to the protein function [29–31]. Since the mutation of amino acids according to the LCD analysis or AvNAPSA score could damage polypeptide regions critical to protein function, we utilized conservation scores from the Consurf webserver results. Now, when a residue had a conservation score higher than 5, it was considered important to protein function and excluded from possible mutation by LCD analysis results.

### Final PySupercharge algorithm

**Fig. 2:**
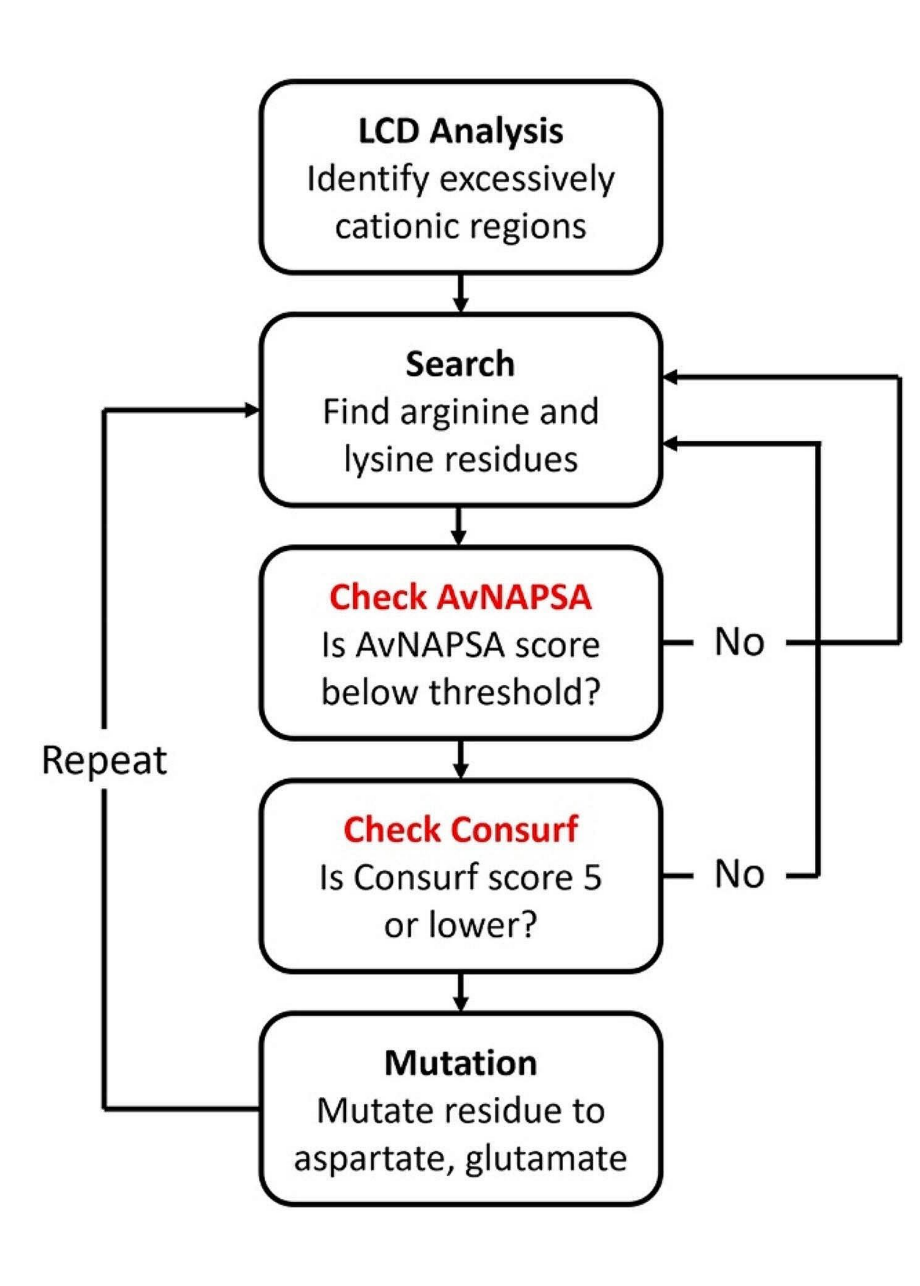
Logic scheme of PySupercharge. How the PySupercharge algorithm supercharges protein sequences. PySupercharge takes amino acids’ AvNAPSA and ConSurf scores into account before mutating them

The final version of our PySupercharge algorithm incorporates both LCD analysis and protein function/stability loss minimization methods (Fig. 2). Firstly, the program accepts the Protein Database (.pdb extension file; ‘PDB format’) and Consurf files (consurf_grades.txt; obtained by running process at Consurf website) for the protein of interest. Secondly, it operates LCD analysis with the established window size. If a cationic supercharged region is identified, Arg and Lys residues in that region are found and remembered. Thirdly, the program calculates the AvNAPSA score of such flagged residues and stores those that have a score in the user-defined range. Then, the algorithm sorts these residues based on the Consurf score so that only those with a score less than or equal to 5 (hence, highly variable and deemed functionally unimportant) are considered for mutation. Finally, the actual mutations from the approved Arg and Lys residues to Glu or Asp residues are carried out.

This final product, the PySupercharge algorithm, is available for use on our laboratory webserver, https://mb.re.kr/apps/supercharge/. We included an example of GFP supercharging on the webserver and in an additional file [see Additional file 3].

### Protein expression analysis

We mutated multiple originally non-secretable proteins using both manual and PySupercharge-aided mutation to analyze secretion ability in *fleQ*-knockout *P. fluorescens.* MFP and OMP were co-expressed with the ABC protein in these cells for the secretion of proteins. Nitrocellulose membranes (Amersham, Germany) were used for Western blotting. Polyclonal anti-LARD3 rabbit immunoglobulin G (rIgG) was utilized as the primary antibody with 1:3000 dilution in 5% skim milk solution, and anti-rabbit recombinant goat IgG-peroxidase (anti-rIgG goat IgG-peroxidase) was used as the secondary antibody with 1:5000 dilution. The bands were then detected using a chemiluminescence agent (Advansta WesternBright Pico, San Jose, CA). Western blot images were acquired using an Azure C600 automatic detection system (Azure Biosystems, Dublin, CA). The genes for protein expression were synthesized using Bionics (http://www.bionicsro.co.kr/) gene synthesis service. The C-terminal LARD3 secretion signal of thermostable lipase TliA was appended to every protein secreted in this study, recognized by the *P. fluorescens* TliDEF ABC transporter. (A western blot showing the secreted LARD3-only control is in an additional file [see Additional File 4]) Protein expression was carried out through constitutive expression. The amino acid sequences for all proteins used in this research are in the additional files [see Additional file 1, Additional file 2].

## Results

### Secretion of human growth factors by manual mutation

**Fig. 3:**
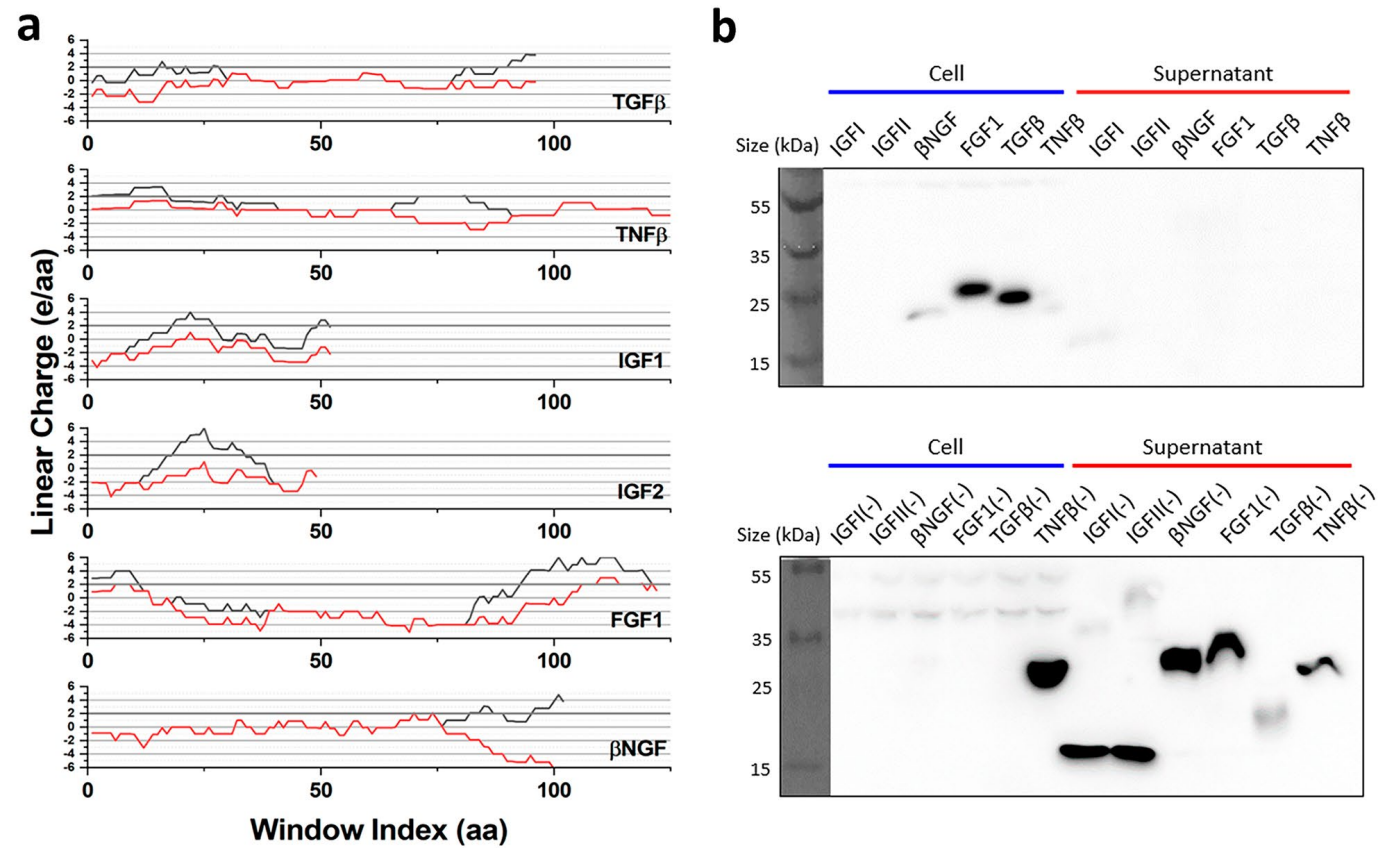
Secretion results of manually supercharged human growth factors. **(a)** LCD analysis results of wildtype sequences (shown in grey) and manually supercharged sequences (shown in red) of various human growth factors. Each plotted point matches an amino acid position to the LCD value of the 20-amino-acid window starting from that position. **(b)** Adapted from “Utilizing the ABC Transporter for Growth Factor Production by *fleQ* deletion mutant of *Pseudomonas fluorescens*”, Fabia et al., *Biomedicines* 2022, 9(6), p. 679 [2]. Adapted with permission- label sizes and colors have been modified. The western blotting results of manually mutated growth factors (bottom row) show enhanced secretion ability compared to the wildtype proteins (top row). A separate high-exposure western blot of wildtype IGF1 and IGF2 is in an additional file [see Additional File 4] due to low visibility in this figure

Various non-secretable human growth factors, namely transforming growth factor beta (TGFβ), fibroblast growth factor I (FGF1), insulin-like growth factor I and II (IGFI and IGFII), and beta-nerve growth factor (βNGF) were mutated manually before the development of PySupercharge and tested for secretion ability. The manual mutation process was simple; cationic supercharged regions were identified through LCD analysis and positive amino acids within the region were converted to negative amino acids. This approach proved successful for short sequences within growth factors but did not take function loss into account and was arduous for larger protein sequences (Fig. 3). We used AlphaFold 2 to estimate the structure of the post-mutation proteins [32]. The AlphaFold2-predicted structures of the proteins before and after mutation are included in an additional file [see Additional File 6].

### Secretion of SARS-CoV-2 spike proteins with PySupercharge

**Fig. 4:**
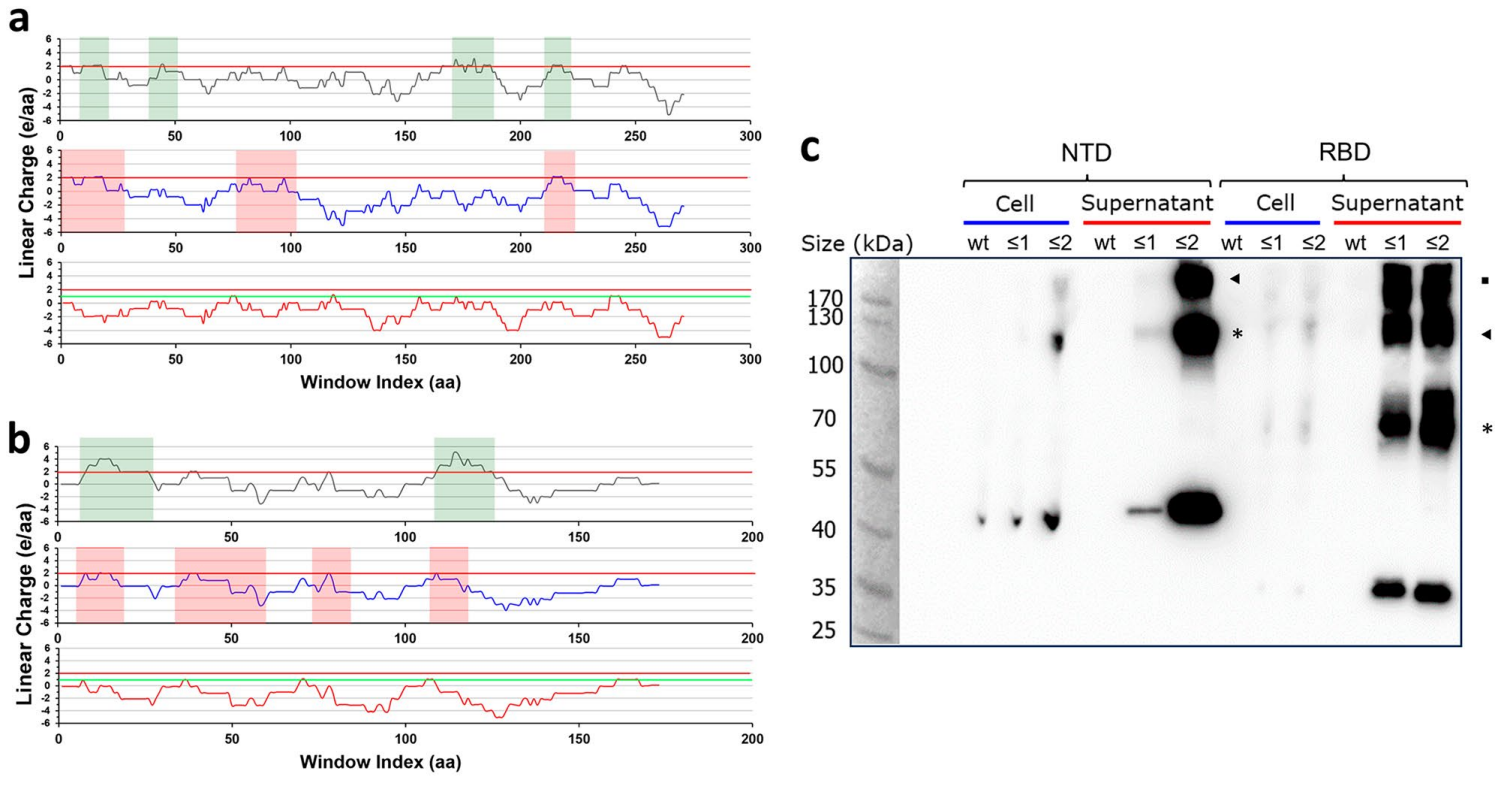
Secretion of automatically supercharged SARS-CoV-2 S1 protein NTD and RBD. **(a)** Top graph: LCD analysis result of wildtype SARS-CoV-2 S1 protein NTD sequence (cationic supercharged regions of LCD > 2 shaded green). Middle graph: Sequence mutated by PySupercharge to make NTD (LCD ≤ 2). Note that all the shaded regions from the top graph have been modified to stay below or on the LCD = 2 reference line. LCD > 1 regions have been shaded in red for comparison with NTD (LCD ≤ 1). Bottom graph: Sequence of NTD (LCD ≤ 1), mutated in a similar manner but with a cutoff value of 1. The LCD values for the SARS-CoV-2 spike protein domains were calculated with histidine charge configuration + 0.1. All of the regions have been mutated to stay below or on the green LCD = 1 reference line. **(b)** Similar set of graphs for SARS-CoV-2 S1 protein RBD. **(c)** The western blot analysis results of the NTD (1st -6th lane) and RBD (7th -12th lane). Supercharged NTD and RBD sequences clearly display greater secretion ability. A separate high-exposure western blot of wildtype RBD is in an additional file [see Additional File 4] due to low visibility in this figure. (*, dimer protein; ▶, trimer protein; ■, tetramer protein)

The two domains of the SARS-CoV-2 spike protein homotrimer S1 (PDB ID 6ZP0) are the N-terminal domain (NTD) and receptor binding domain (RBD). LCD analysis identified two cationic supercharged regions of LCD value over 2 in each domain, which could possibly inhibit bacterial secretion. To investigate the correlation between the linear charge density and secretion ability, both NTD and RBD sequences were negatively supercharged to two LCD cutoff values: ≤ 2 and ≤ 1 (Fig. 4). In the case of the NTD, there was a total of 26 arginine and lysine residues within the coding region. Among these residues, PySupercharge mutated 6 and 5 for NTD and RBD respectively. The AlphaFold2-predicted structures of the wildtype, LCD ≤ 2 and LCD ≤ 1 versions of NTD and RBD are included in an additional file [see Additional File 6].

Analyzing the western blotting results for both the NTD and RBD, supercharged proteins are found in much greater amounts in the supernatant after secretion than the wildtype proteins (Fig. 4c). This implies the greater secretion ability of supercharged proteins. Notably, LCD ≤ 1 cutoff mutant proteins are shown to have considerably better secretion ability than those of the ≤ 2 cutoff value. SDS-PAGE data for NTD and RBD is in an additional file [see Additional File 5].

### Secretion of botulinum toxin light chain (BoNT/A)

Among the 7 serotypes of botulinum toxin, type A is the most widely used in both academia and the therapeutic industry. This neurotoxin is consisted of a heavy chain that enables internalization of the toxin into the presynaptic terminal and a light chain that cleaves synaptosome-associated proteins (SNAP25) [33]. LCD analysis of the light chain, BoNT/A, identified 6 cationic supercharged regions with the LCD greater than 2. Two mutants, BoNT (LCD ≤ 2) and BoNT (LCD ≤ 1), were created with the LCD cutoff value of 2 and 1 respectively using PySupercharge. BoNT (LCD ≤ 1) was mutated with stricter criteria to compare the secretion ability. The AlphaFold2-predicted structures of BoNT, BoNT (LCD ≤ 2) and BoNT (LCD ≤ 1) are included in an additional file [see Additional File 6].

**Fig. 5:**
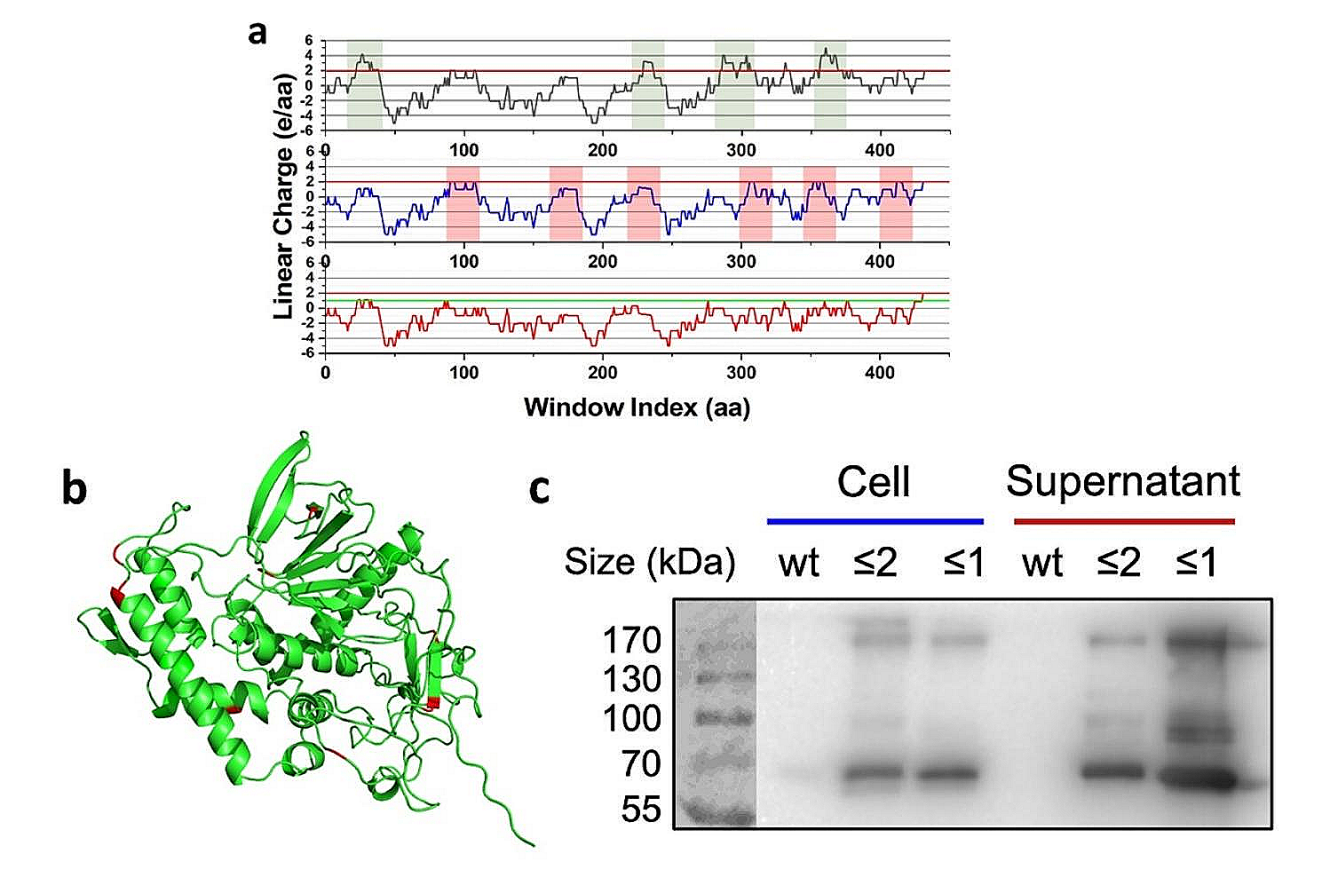
Secretion of supercharged botulinum toxin type A. **(a)** Top graph: LCD analysis result of BoNT wildtype sequence (cationic supercharged regions of LCD > 2 shaded green). Middle graph: Mutated sequence of BoNT (LCD ≤ 2). Note that all the shaded regions from the top graph have been modified to stay below or on the red LCD = 2 reference line. LCD > 1 regions have been shaded in red for easy comparison with BoNT (LCD ≤ 1). Bottom graph: Mutated sequence of BoNT (LCD ≤ 1). Note that all the shaded regions from the top graph have been modified to stay below or on the green LCD = 1 reference line. **(b)** BoNT/A wildtype protein structure with residues mutated in BoNT (LCD ≤ 2) highlighted in red. **(c)** The western blot analysis results of BoNT. Supercharged BoNT (LCD ≤ 2) and BoNT (LCD ≤ 1) clearly display higher secretion ability, the latter being even higher than the former

The Western blotting results for BoNT in the cell and supernatant show that the secretion ability of both of the two supercharged proteins is much greater than that of the wildtype protein (Fig. 5). The LCD ≤ 1 cutoff mutant performs even better than the ≤ 2 mutant, similar to the SARS-CoV-2 spike protein analysis. SDS-PAGE data for BoNT is in an additional file [see Additional File 5].

## Discussion

Protein supercharging can improve secretion of proteins, enabling them to be produced through bacterial secretion systems using ABC transporters. In most previous supercharging-related studies, the focus was on the surface charge of the protein [28, 34]. We focused instead on the charge of the unfolded polypeptide chain, modifying it to aid in its secretion. In our previous study, we defined a new criterion for protein supercharging, the linear charge density. Based on the LCD calculations, protein sequences were manually mutated and tested for secretion ability. Then we took a step further, automating the LCD analysis and mutation process. We also implemented two separate tools, AvNAPSA and Consurf, in our LCD analysis.

AvNAPSA and Consurf are two analysis processes utilized to prevent unwanted effects from mutation by LCD analysis. Since mutation using only LCD analysis is entirely dependent on amino acid charge, structural stability or functionality could be greatly disrupted by mutation. AvNAPSA was originally designed for finding and mutating surface residues to manipulate protein surface charge [27]. We translated it to Python and used it to separate the surface and inner residues, now only mutating these surface residues to minimize the destabilization of protein structure by high mutation load. Consurf traces the evolutionary history of the residues and returns the conservation score [29–31]. A residue with a high conservation score has a higher chance of being critical to protein function, and therefore is exempt from mutation.

PySupercharge aims to minimize protein function loss through exclusion of evolutionarily conserved residues from mutation. However, it does not guarantee preservation of protein activity. Additionally, our present algorithm does not consider the types and significance of the chemical bonds formed around particular amino acid residues. So, we risk mutating a surface residue that had formed important hydrogen bonds when it was present. This could result in the loss of structural integrity. Therefore, a potential direction for further development of the algorithm would be to refine the mutation candidate selection to residues not engaging in hydrogen bonding with others. As for the current algorithm, we have added an option for excluding user-selected residues in the supercharging process by their indexes (e.g., a user input of ‘35, 136’ in the Exclusion field prevents the 35th and 136th residues from being mutated). Users may choose to exclude certain residues if they are predicted to be functionally significant.

Overall, we have developed PySupercharge, a one-click tool to mutate amino acid sequences based on linear charge density analysis. Given the PDB and Consurf files of the target protein, PySupercharge can also calculate AvNAPSA and Consurf scores and use them to maintain structural stability and exclude functionally important residues from mutation, respectively. We verified that non-secretable protein sequences mutated with PySupercharge showed increased secretion efficiency, and so suggest this tool for future research in effective protein secretion. Our tool is available on the webserver https://mb.re.kr/apps/supercharge/.

### Electronic supplementary material

Below is the link to the electronic supplementary material.

Additional file 1: Amino acid sequences of wildtype proteins expressed. Sequences of all of the wildtype proteins we expressed and secreted in the study.

Additional file 2: Amino acid sequences of negatively supercharged proteins. Sequences of all of the supercharged proteins (either manually or by PySupercharge) we expressed and secreted in the study.

Additional file 3: Example negative supercharging and LCD analysis of green fluorescent protein (GFP). Amino acid sequences for wildtype GFP (upper text) and negatively supercharged GFP (lower text). Four amino acids have been mutated. LCD analysis graph of the same (wildtype: blue, LCD ≤ 2: orange). A 3D model of the wildtype GFP with AvNAPSA <150 residues highlighted in red is shown below.

Additional file 4: High-exposure western blotting images of wildtype proteins. High-exposure western blotting images of wildtype IGF1, IGF2, and SARS-CoV-2 RBD.

Additional file 5: SDS-PAGE images of SARS-CoV-2 domains and BoNT. SDS-PAGE data for supercharged SARS-CoV-2 domains and BoNT in the study.

Additional File 6: AlphaFold2-generated structures of wildtype and supercharged proteins. Superimposed AlphaFold2-generated protein structures of all proteins in the study.

## Data Availability

No datasets were generated or analysed during the current study.
