# Supplementary material for: PySupercharge: a python algorithm for enabling ABC transporter bacterial secretion of all proteins through amino acid mutation
Source: Microb Cell Fact. 2024 Apr 20;23:115. doi: 10.1186/s12934-024-02342-z (PMC11031901; doi:10.1186/s12934-024-02342-z)
Supplement: Supplementary file 1 — Additional file 1: Amino acid sequences of wildtype proteins expressed. Sequences of all of the wildtype proteins we expressed and secreted in the study. [file 12934_2024_2342_MOESM1_ESM.docx]

**>TGFβ**MSRHHHHHHMSRALDTNYCFSSTEKNCCVRQLYIDFRKDLGWKWIHEPKGYHANFCLGPCPYIWSLDTQYSKVLALYNQHNPGASAAPCCVPQALEPLPIVYYVGRKPKVEQLSNMIVRSCKCSELIEGRGSDGNDLIQGGKGADFIEGGKGNDTIRDNSGHNTFLFSGHFGQDRIIGYQPTDRLVFQGADGSTDLRDHAKAVGADTVLSFGADSVTLVGVGLGGLWSEGVLIS

Expected MW: 25742.00

**>TNFβ**MSRHHHHHHMLPGVGLTPSAAQTARQHPKMHLAHSTLKPAAHLIGDPSKQNSLLWRANTDRAFLQDGFSLSNNSLLVPTSGIYFVYSQVVFSGKAYSPKATSSPLYLAHEVQLFSSQYPFHVPLLSSQKMVYPGLQEPWLHSMYHGAAFQLTQGDQLSTHTDGIPHLVLSPSTVFFGAFALELIEGRGSDGNDLIQGGKGADFIEGGKGNDTIRDNSGHNTFLFSGHFGQDRIIGYQPTDRLVFQGADGSTDLRDHAKAVGADTVLSFGADSVTLVGVGLGGLWSEGVLIS

Expected MW: 31365.16

**>IGF1**MSRHHHHHHMGPETLCGAELVDALQFVCGDRGFYFNKPTGYGSSSRRAPQTGIVDECCFRSCDLRRLEMYCAPLKPAKSAELIEGRGSDGNDLIQGGKGADFIEGGKGNDTIRDNSGHNTFLFSGHFGQDRIIGYQPTDRLVFQGADGSTDLRDHAKAVGADTVLSFGADSVTLVGVGLGGLWSEGVLIS

Expected MW: 20358.66

**>IGF2**MSRHHHHHHMAYRPSETLCGGELVDTLQFVCGDRGFYFSRPASRVSRRSRGIVEECCFRSCDLALLETYCATPAKSEELIEGRGSDGNDLIQGGKGADFIEGGKGNDTIRDNSGHNTFLFSGHFGQDRIIGYQPTDRLVFQGADGSTDLRDHAKAVGADTVLSFGADSVTLVGVGLGGLWSEGVLIS

Expected MW: 20179.38

**>FGF1**MSRHHHHHHMFNLPPGNYKKPKLLYCSNGGHFLRILPDGTVDGTRDRSDQHIQLQLSAESVGEVYIKSTETGQYLAMDTDGLLYGSQTPNEECLFLERLEENHYNTYISKKHAEKNWFVGLKKNGSCKRGPRTHYGQKAILFLPLPVSSDELIEGRGSDGNDLIQGGKGADFIEGGKGNDTIRDNSGHNTFLFSGHFGQDRIIGYQPTDRLVFQGADGSTDLRDHAKAVGADTVLSFGADSVTLVGVGLGGLWSEGVLIS

Expected MW: 28539.78

**>βNGF**MSRHHHHHHMSSSHPIFHRGEFSVCDSVSVWVGDKTTATDIKGKEVMVLGEVNINNSVFKQYFFETKCRDPNPVDSGCRGIDSKHWNSYCTTTHTFVKALTMDGKQAAWRFIRIDTACVCVLSRKAVRRAELIEGRGSDGNDLIQGGKGADFIEGGKGNDTIRDNSGHNTFLFSGHFGQDRIIGYQPTDRLVFQGADGSTDLRDHAKAVGADTVLSFGADSVTLVGVGLGGLWSEGVLIS

Expected MW: 26198.30

**>SARS-CoV-2 S1 NTD**MSRHHHHHHVNLTTRTQLPPAYTNSFTRGVYYPDKVFRSSVLHSTQDLFLPFFSNVTWFHAIHVSGTNGTKRFDNPVLPFNDGVYFASTEKSNIIRGWIFGTTLDSKTQSLLIVNNATNVVIKVCEFQFCNDPFLGVYYHKNNKSWMESEFRVYSSANNCTFEYVSQPFLMDLEGKQGNFKNLREFVFKNIDGYFKIYSKHTPINLVRDLPQGFSALEPLVDLPIGINITRFQTLLALHRSYLTPGDSSSGWTAGAAAYYVGYLQPRTFLLKYNENGTITDAVDCALDPLSETKCTLKSELIEGRGSDGNDLIQGGKGADFIEGGKGNDTIRDNSGHNTFLFSGHFGQDRIIGYQPTDRLVFQGADGSTDLRDHAKAVGADTVLSFGADSVTLVGVGLGGLWSEGVLIS

Expected MW: 45479.02

**>SARS-CoV-2 S1 RBD**MSRHHHHHHPNITNLCPFGEVFNATRFASVYAWNRKRISNCVADYSVLYNSASFSTFKCYGVSPTKLNDLCFTNVYADSFVIRGDEVRQIAPGQTGKIADYNYKLPDDFTGCVIAWNSNNLDSKVGGNYNYLYRLFRKSNLKPFERDISTEIYQAGSTPCNGVEGFNCYFPLQSYGFQPTNGVGYQPYRVVVLSFELLHAPELIEGRGSDGNDLIQGGKGADFIEGGKGNDTIRDNSGHNTFLFSGHFGQDRIIGYQPTDRLVFQGADGSTDLRDHAKAVGADTVLSFGADSVTLVGVGLGGLWSEGVLIS

Expected MW: 34224.05

**>BoNT/A**MSRHHHHHHEFGSMEFVNKQFNYKDPVNGVDIAYIKIPNAGQMQPVKAFKIHNKIWVIPERDTFTNPEEGDLNPPPEAKQVPVSYYDSTYLSTDNEKDNYLKGVTKLFERIYSTDLGRMLLTSIVRGIPFWGGSTIDTELKVIDTNCINVIQPDGSYRSEELNLVIIGPSADIIQFECKSFGHEVLNLTRNGYGSTQYIRFSPDFTFGFEESLEVDTNPLLGAGKFATDPAVTLAHELIHAGHRLYGIAINPNRVFKVNTNAYYEMSGLEVSFEELRTFGGHDAKFIDSLQENEFRLYYYNKFKDIASTLNKAKSIVGTTASLQYMKNVFKEKYLLSEDTSGKFSVDKLKFDKLYKMLTEIYTEDNFVKFFKVLNRKTYLNFDKAVFKINIVPKVNYTIYDGFNLRNTNLAANFNGQNTEINNMNFTKLKNFTGLFEFYKLLCVDGIITSKTKSLIEGRGSDGNDLIQGGKGADFIEGGKGNDTIRDNSGHNTFLFSGHFGQDRIIGYQPTDRLVFQGADGSTDLRDHAKAVGADTVLSFGADSVTLVGVGLGGLWSEGVLIS

Expected MW: 63327.43
