# Supplementary material for: PySupercharge: a python algorithm for enabling ABC transporter bacterial secretion of all proteins through amino acid mutation
Source: Microb Cell Fact. 2024 Apr 20;23:115. doi: 10.1186/s12934-024-02342-z (PMC11031901; doi:10.1186/s12934-024-02342-z)
Supplement: Supplementary file 2 — Additional file 2: Amino acid sequences of negatively supercharged proteins. Sequences of all of the supercharged proteins (either manually or by PySupercharge) we expressed and secreted in the study. [file 12934_2024_2342_MOESM2_ESM.docx]

**>Negatively supercharged TGFβ**MSRHHHHHHMSRALDTNYCFSSTEENCCVRQLYIDFREDLGWKWIHEPKGYHANFCLGPCPYIWSLDTQYSKVLALYNQHNPGASAAPCCVPQALEPLPIVYYVGRDPKVEQLSNMIVDSCKCSELIEGRGSDGNDLIQGGKGADFIEGGKGNDTIRDNSGHNTFLFSGHFGQDRIIGYQPTDRLVFQGADGSTDLRDHAKAVGADTVLSFGADSVTLVGVGLGGLWSEGVLIS

Expected MW: 25689.70

**>Negatively supercharged TNFβ**MSRHHHHHHMLPGVGLTPSAAQTAQQHPQMHLAHSTLKPAAHLIGDPSTQNSLLWRANTDRAFLQDGFSLSNNSLLVPTSGIYFVYSQVVFSGEAYSPEATSSPLYLAHEVQLFSSQYPFHVPLLSSQKMVYPGLQEPWLHSMYHGAAFQLTQGDQLSTHTDGIPHLVLSPSTVFFGAFALELIEGRGSDGNDLIQGGKGADFIEGGKGNDTIRDNSGHNTFLFSGHFGQDRIIGYQPTDRLVFQGADGSTDLRDHAKAVGADTVLSFGADSVTLVGVGLGGLWSEGVLIS

Expected MW: 31311.8

**>Negatively supercharged IGF1**MSRHHHHHHMGPETLCGAELVDALQFVCGDRGFYFNEPTGYGSSSQRAPQTGIVDECCFRSCDLQQLEMYCAPLEPAKSAELIEGRGSDGNDLIQGGKGADFIEGGKGNDTIRDNSGHNTFLFSGHFGQDRIIGYQPTDRLVFQGADGSTDLRDHAKAVGADTVLSFGADSVTLVGVGLGGLWSEGVLIS

Expected MW: 20276.37

**>Negatively supercharged IGF2**MSRHHHHHHMAYRPSETLCGGELVDTLQFVCGDRGFYFSEPASQVSQQSRGIVEECCFRSCDLALLETYCATPAKSELIEGRGSDGNDLIQGGKGADFIEGGKGNDTIRDNSGHNTFLFSGHFGQDRIIGYQPTDRLVFQGADGSTDLRDHAKAVGADTVLSFGADSVTLVGVGLGGLWSEGVLIS

Expected MW: 19939.02

**>Negatively supercharged FGF1**MSRHHHHHHMFNLPPGNYQQPKLLYCSNGGHFLRILPDGTVDGTRDDSDQHIQLQLSAESVGEVYIKSTETGQYLAMDTDGLLYGSQTPNEECLFLERLEENHYNTYISQEHAEQNWFVGLKQNGSCKDGPRTHYGQKAILFLPLPVSSDELIEGRGSDGNDLIQGGKGADFIEGGKGNDTIRDNSGHNTFLFSGHFGQDRIIGYQPTDRLVFQGADGSTDLRDHAKAVGADTVLSFGADSVTLVGVGLGGLWSEGVLIS

Expected MW: 28458.31

**>Negatively supercharged βNGF**MSRHHHHHHMSSSHPIFHRGEFSVCDSVSVWVGDKTTATDIKGKEVMVLGEVNINNSVFKQYFFETKCRDPNPVDSGCRGIDSKHWNSYCTTTHTFVKALTMDGEQAAWDFIDIDTACVCVLSEEAVEEAELIEGRGSDGNDLIQGGKGADFIEGGKGNDTIRDNSGHNTFLFSGHFGQDRIIGYQPTDRLVFQGADGSTDLRDHAKAVGADTVLSFGADSVTLVGVGLGGLWSEGVLIS

Expected MW: 26036.77

**>Negatively supercharged SARS-CoV-2 S1 NTD (LCD ≤ 2)**
MSRHHHHHHVNLTTRTQLPPAYTNSFTRGVYYPDKVFRSSVLHSTQDLFLPFFSNVTWFHAIHVSGTNGTDRFDNPVLPFNDGVYFASTEKSNIIRGWIFGTTLDSKTQSLLIVNNATNVVIKVCEFQFCNDPFLGVYYHDNNESWMESEFRVYSSANNCTFEYVSQPFLMDLEGKQGNFKNLREFVFENIDGYFKIYSDHTPINLVRDLPQGFSALEPLVDLPIGINITRFQTLLALHRSYLTPGDSSSGWTAGAAAYYVGYLQPRTFLLEYNENGTITDAVDCALDPLSETKCTLKSELIEGRGSDGNDLIQGGKGADFIEGGKGNDTIRDNSGHNTFLFSGHFGQDRIIGYQPTDRLVFQGADGSTDLRDHAKAVGADTVLSFGADSVTLVGVGLGGLWSEGVLIS

Expected MW: 45442.59

**>Negatively supercharged SARS-CoV-2 S1 NTD (LCD ≤ 1)**MSRHHHHHHVNLTTRTQLPPAYTNSFTDGVYYPDKVFDSSVLHSTQDLFLPFFSNVTWFHAIHVSGTNGTDRFDNPVLPFNDGVYFASTEKSNIIRGWIFGTTLDSDTQSLLIVNNATNVVIKVCEFQFCNDPFLGVYYHKNNKSWMESEFDVYSSANNCTFEYVSQPFLMDLEGKQGNFKNLREFVFDNIDGYFKIYSKHTPINLVDDLPQGFSALEPLVDLPIGINITRFQTLLALHDSYLTPGDSSSGWTAGAAAYYVGYLQPRTFLLDYNENGTITDAVDCALDPLSETKCTLKSELIEGRGSDGNDLIQGGKGADFIEGGKGNDTIRDNSGHNTFLFSGHFGQDRIIGYQPTDRLVFQGADGSTDLRDHAKAVGADTVLSFGADSVTLVGVGLGGLWSEGVLIS

Expected MW: 45221.18

**>Negatively supercharged SARS-CoV-2 S1 RBD (LCD ≤ 2)**MSRHHHHHHPNITNLCPFGEVFNATRFASVYAWNRKDISNCVADYSVLYNSASFSTFKCYGVSPTKLNDLCFTNVYADSFVIRGDEVRQIAPGQTGKIADYNYKLPDDFTGCVIAWNSNNLDSKVGGNYNYLYRLFRDSNLEPFERDISTEIYQAGSTPCNGVEGFNCYFPLQSYGFQPTNGVGYQPYRVVVLSFELLHAPELIEGRGSDGNDLIQGGKGADFIEGGKGNDTIRDNSGHNTFLFSGHFGQDRIIGYQPTDRLVFQGADGSTDLRDHAKAVGADTVLSFGADSVTLVGVGLGGLWSEGVLIS

Expected MW: 34170.81

**>Negatively supercharged SARS-CoV-2 S1 RBD (LCD ≤ 1)**MSRHHHHHHPNITNLCPFGEVFNATRFASVYAWNRDDISNCVADYSVLYNSASFSTFKCYGVSPTDLNDLCFTNVYADSFVIRGDEVRQIAPGQTGKIADYNYDLPDDFTGCVIAWNSNNLDSKVGGNYNYLYRLFDDSNLDPFERDISTEIYQAGSTPCNGVEGFNCYFPLQSYGFQPTNGVGYQPYRVVVLSFELLHAPELIEGRGSDGNDLIQGGKGADFIEGGKGNDTIRDNSGHNTFLFSGHFGQDRIIGYQPTDRLVFQGADGSTDLRDHAKAVGADTVLSFGADSVTLVGVGLGGLWSEGVLIS

Expected MW: 34076.43

**>Negatively supercharged BoNT/A (LCD ≤ 2)**
MSRHHHHHHEFGSMEFVNKQFNYKDPVNGVDIAYIEIPNAGQMQPVKAFKIHNDIWVIPERDTFTNPEEGDLNPPPEAKQVPVSYYDSTYLSTDNEKDNYLKGVTKLFERIYSTDLGRMLLTSIVRGIPFWGGSTIDTELKVIDTNCINVIQPDGSYRSEELNLVIIGPSADIIQFECKSFGHEVLNLTRNGYGSTQYIRFSPDFTFGFEESLEVDTNPLLGAGKFATDPAVTLAHELIHAGHRLYGIAINPNRVFDVNTNAYYEMSGLEVSFEELRTFGGHDAKFIDSLQENEFRLYYYNKFKDIASTLNEAESIVGTTASLQYMKNVFKEKYLLSEDTSGKFSVDKLKFDKLYEMLTEIYTEDNFVKFFKVLNRKTYLNFDDAVFEINIVPKVNYTIYDGFNLRNTNLAANFNGQNTEINNMNFTKLKNFTGLFEFYKLLCVDGIITSKTKSLIEGRGSDGNDLIQGGKGADFIEGGKGNDTIRDNSGHNTFLFSGHFGQDRIIGYQPTDRLVFQGADGSTDLRDHAKAVGADTVLSFGADSVTLVGVGLGGLWSEGVLIS

Expected MW: 63292.88

**>Negatively supercharged BoNT/A (LCD ≤ 1)**MSRHHHHHHEFGSMEFVNKQFNYKDPVNGVDIAYIEIPNAGQMQPVKAFKIHNDIWVIPERDTFTNPEEGDLNPPPEAKQVPVSYYDSTYLSTDNEKDNYLKGVTKLFERIYSTDLGEMLLTSIVRGIPFWGGSTIDTELKVIDTNCINVIQPDGSYRSEELNLVIIGPSADIIQFECKSFGHEVLNLTDNGYGSTQYIRFSPDFTFGFEESLEVDTNPLLGAGKFATDPAVTLAHELIHAGHRLYGIAINPNEVFEVNTNAYYEMSGLEVSFEELRTFGGHDAKFIDSLQENEFRLYYYNKFKDIASTLNDAESIVGTTASLQYMKNVFDEKYLLSEDTSGKFSVDKLKFDKLYEMLTEIYTEDNFVKFFKVLNRDTYLNFDEAVFKINIVPKVNYTIYDGFNLENTNLAANFNGQNTEINNMNFTKLENFTGLFEFYKLLCVDGIITSKTKSLIEGRGSDGNDLIQGGKGADFIEGGKGNDTIRDNSGHNTFLFSGHFGQDRIIGYQPTDRLVFQGADGSTDLRDHAKAVGADTVLSFGADSVTLVGVGLGGLWSEGVLIS

Expected MW: 63158.42
