# Supplementary material for: PySupercharge: a python algorithm for enabling ABC transporter bacterial secretion of all proteins through amino acid mutation
Source: Microb Cell Fact. 2024 Apr 20;23:115. doi: 10.1186/s12934-024-02342-z (PMC11031901; doi:10.1186/s12934-024-02342-z)

**> GFP (PDB ID 1GFL)**

ASKGEELFTG VVPILVELDG DVNGHKFSVS GEGEGDATYG KLTLKFICTT
GKLPVPWPTL VTTFSYGVQC FSRYPDHMKR HDFFKSAMPE GYVQERTIFF
KDDGNYKTRA EVKFEGDTLV NRIELKGIDF KEDGNILGHK LEYNYNSHNV
YIMADKQKNG IKVNFKIRHN IEDGSVQLAD HYQQNTPIGD GPVLLPDNHY
LSTQSALSKD PNEKRDHMVL LEFVTAAGIT HGMDELYK

**> Negatively supercharged GFP (LCD ≤ 2): Mutated residues highlighted in cyan**

ASKGEELFTG VVPILVELDG DVNGHKFSVS GEGEGDATYG KLTLKFICTT
GDLPVPWPTL VTTFSYGVQC FSRYPDHMKE HDFFKSAMPE GYVQERTIFF
KDDGNYKTRA EVKFEGDTLV NRIELKGIDF KEDGNILGHK LEYNYNSHNV
YIMADKQKNG IEVNFKIEHN IEDGSVQLAD HYQQNTPIGD GPVLLPDNHY
LSTQSALSKD PNEKRDHMVL LEFVTAAGIT HGMDELYK

**LCD analysis graph:**

**
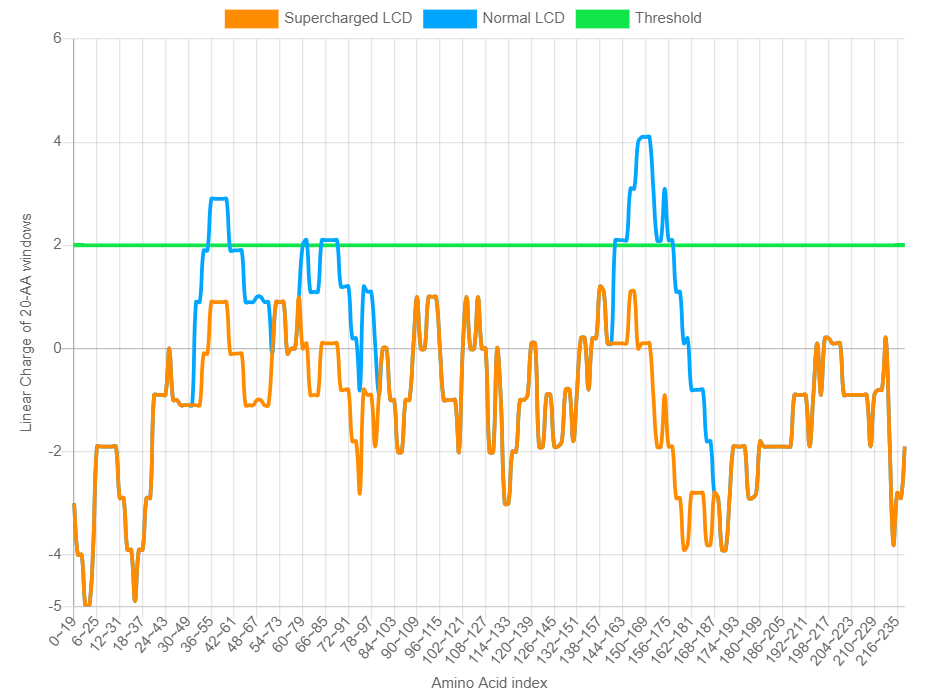
**

**3D Model of Wildtype GFP (AvNAPSA-exposed residues highlighted in red):**


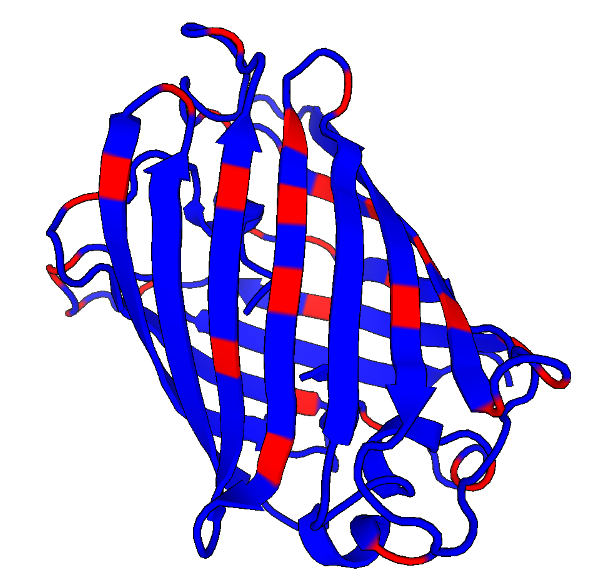

Supplement: Supplementary file 3 — Additional file 3: Example negative supercharging and LCD analysis of green fluorescent protein (GFP). Amino acid sequences for wildtype GFP (upper text) and negatively supercharged GFP (lower text). Four amino acids have been mutated. LCD analysis graph of the same (wildtype: blue, LCD ≤ 2: orange). A 3D model of the wildtype GFP with AvNAPSA <150 residues highlighted in red is shown below. [file 12934_2024_2342_MOESM3_ESM.docx]
