# Supplementary material for: PySupercharge: a python algorithm for enabling ABC transporter bacterial secretion of all proteins through amino acid mutation
Source: Microb Cell Fact. 2024 Apr 20;23:115. doi: 10.1186/s12934-024-02342-z (PMC11031901; doi:10.1186/s12934-024-02342-z)
Supplement: Supplementary file 4 — Additional file 4: High-exposure western blotting images of wildtype proteins. High-exposure western blotting images of wildtype IGF1, IGF2, and SARS-CoV-2 RBD. [file 12934_2024_2342_MOESM4_ESM.docx]

High-exposure Western Blot of IGF1, IGF2 growth factors (Addition to main text Figure 3)


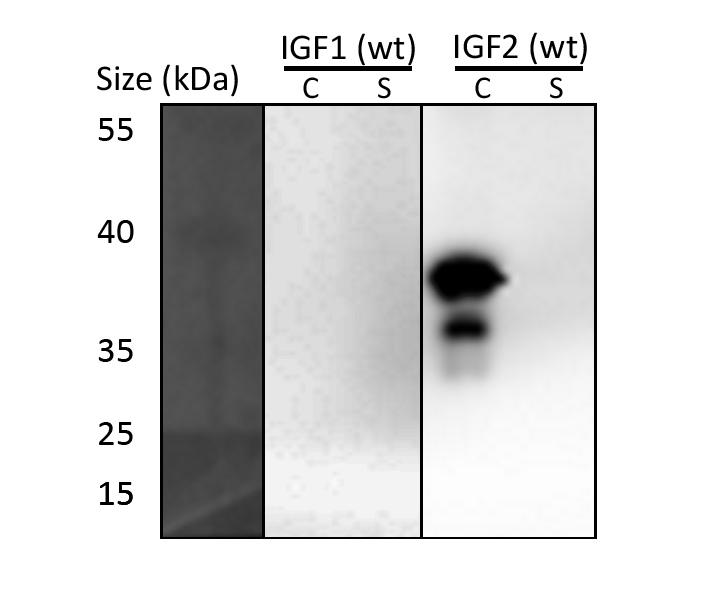


IGF1 wildtype expression/secretion was not observed in the western blot. (Expected MW: 20.4kDa)
IGF2 seems to have been expressed as a dimer. (Expected MW of monomer: 20.2kDa)

High-exposure Western Blot of SARS-CoV-2 RBD domain (Addition to main text Figure 4)


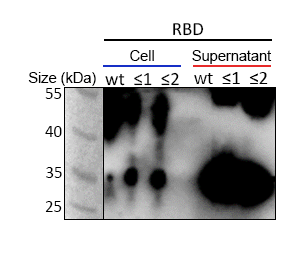


Secretion signal-only (LARD3-only) control Western Blot (supernatant)


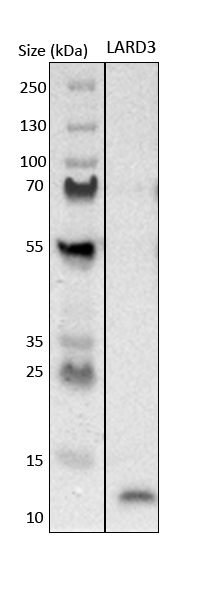


Expected MW of LARD3: 12.4kDa
