# Supplementary material for: PySupercharge: a python algorithm for enabling ABC transporter bacterial secretion of all proteins through amino acid mutation
Source: Microb Cell Fact. 2024 Apr 20;23:115. doi: 10.1186/s12934-024-02342-z (PMC11031901; doi:10.1186/s12934-024-02342-z)
Supplement: Supplementary file 5 — Additional file 5: SDS-PAGE images of SARS-CoV-2 domains and BoNT. SDS-PAGE data for supercharged SARS-CoV-2 domains and BoNT in the study. [file 12934_2024_2342_MOESM5_ESM.docx]

*All SDS-PAGE data was created from the culture supernatant without concentration or protein purification processes.*

SDS-PAGE image of SARS-CoV-2 NTD ≤ 2, RBD ≤ 1, RBD ≤ 2
(Addition to main text Figure 4)

* NTD ≤ 1 data was not included due to no band on SDS-PAGE. The wildtype of both domains and
NTD ≤ 1 showed little expression and displayed no clearly visible band on SDS-PAGE experiments.


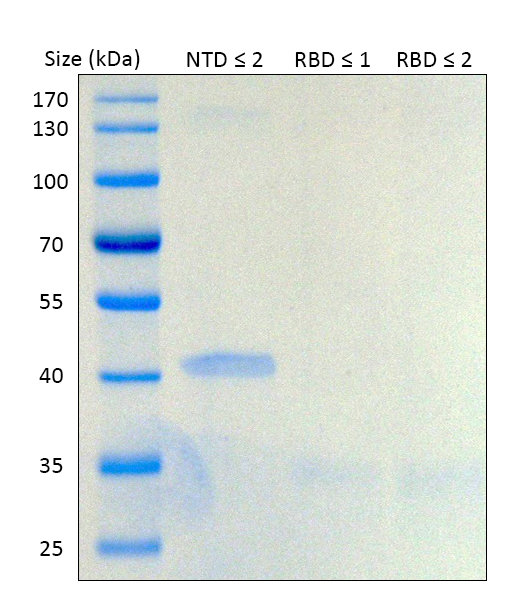


Expected MW of NTD ≤ 2: 45442.59
Expected MW of RBD ≤ 1: 34076.43
Expected MW of RBD ≤ 2: 34170.81

SDS-PAGE image of BoNT ≤ 1, BoNT ≤ 2
(Wildtype expression low; no band visible. Addition to main text Figure 5)


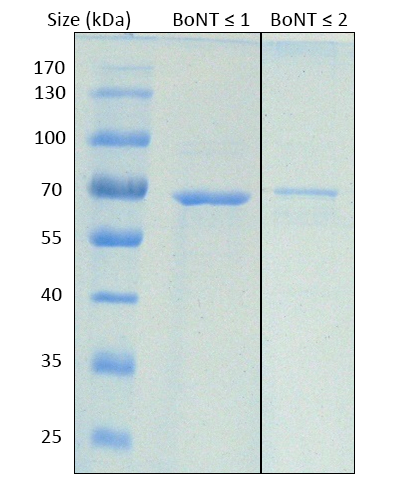

Expected MW of BoNT ≤ 1: 63158.42
Expected MW of BoNT ≤ 2: 63292.88
