# Supplementary material for: PySupercharge: a python algorithm for enabling ABC transporter bacterial secretion of all proteins through amino acid mutation
Source: Microb Cell Fact. 2024 Apr 20;23:115. doi: 10.1186/s12934-024-02342-z (PMC11031901; doi:10.1186/s12934-024-02342-z)

Superimposition of TGFβ, TGFβ (-)


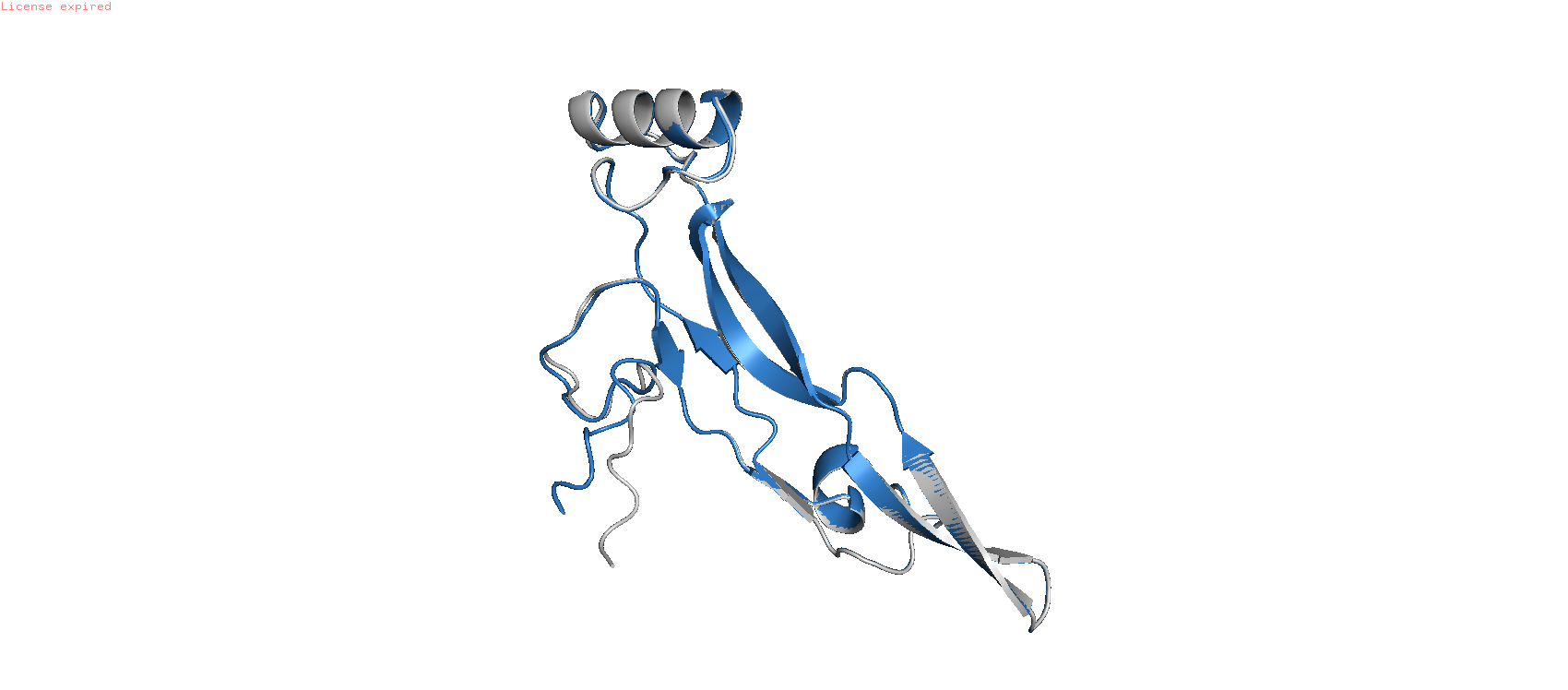


Superimposition of TNFβ, TNFβ (-)


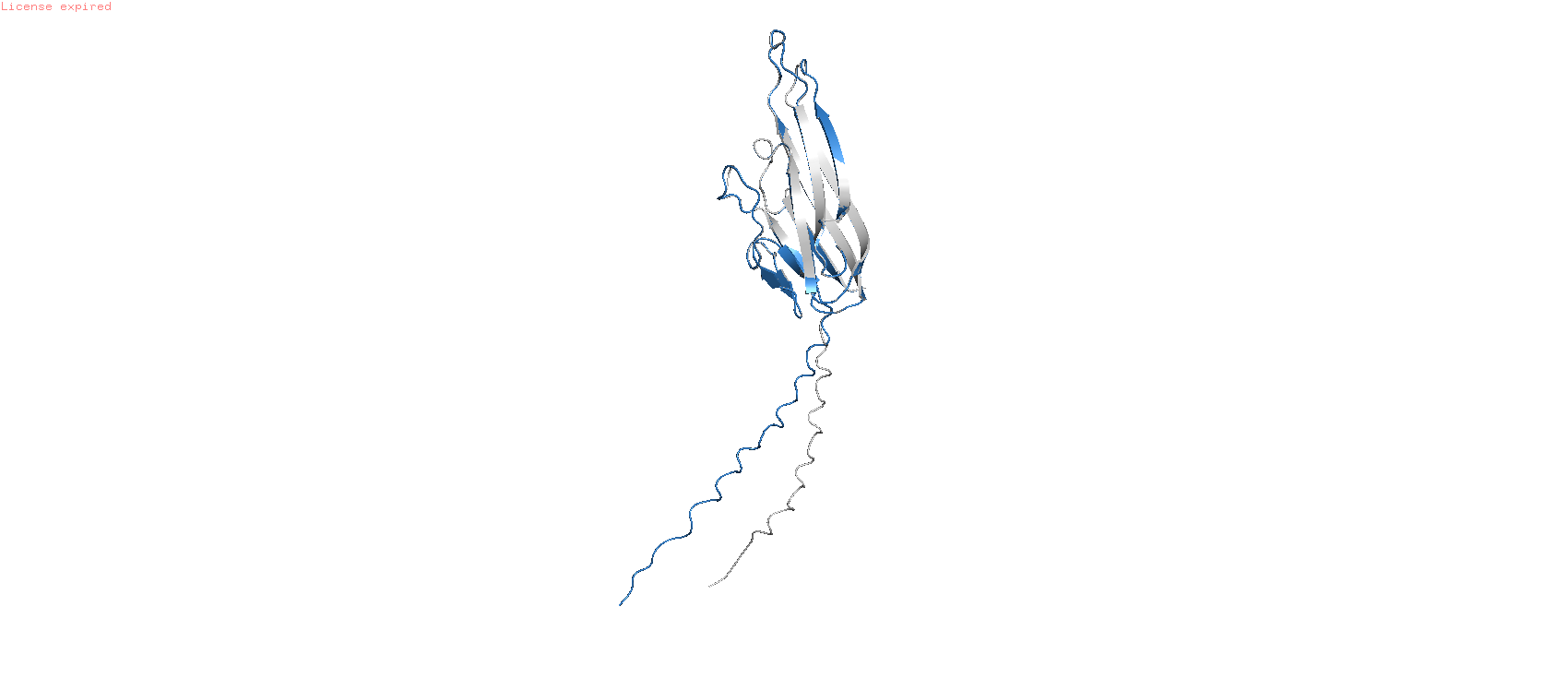


Superimposition of IGF1, IGF1 (-)


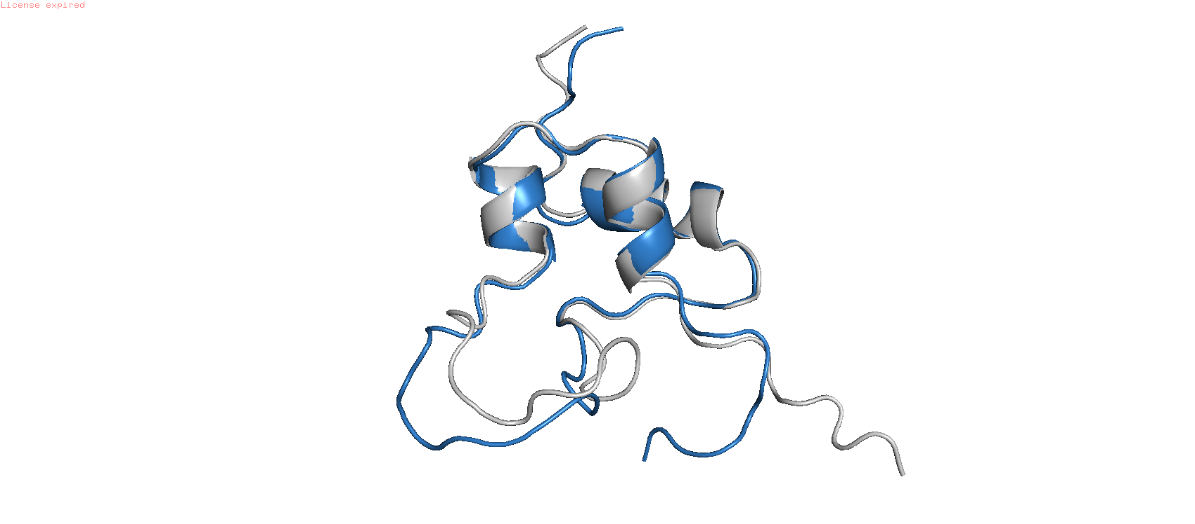


Superimposition of IGF2, IGF2 (-)


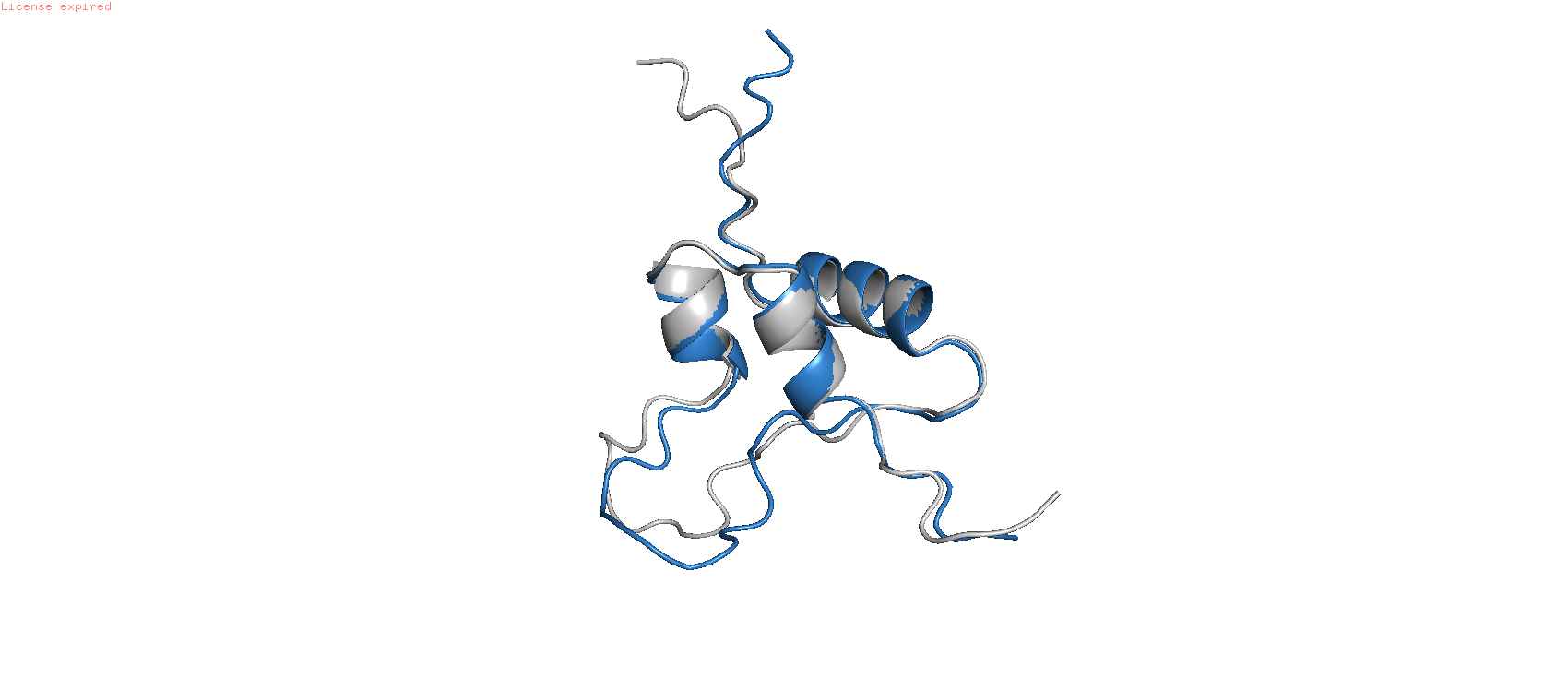


Superimposition of FGF1, FGF1 (-)


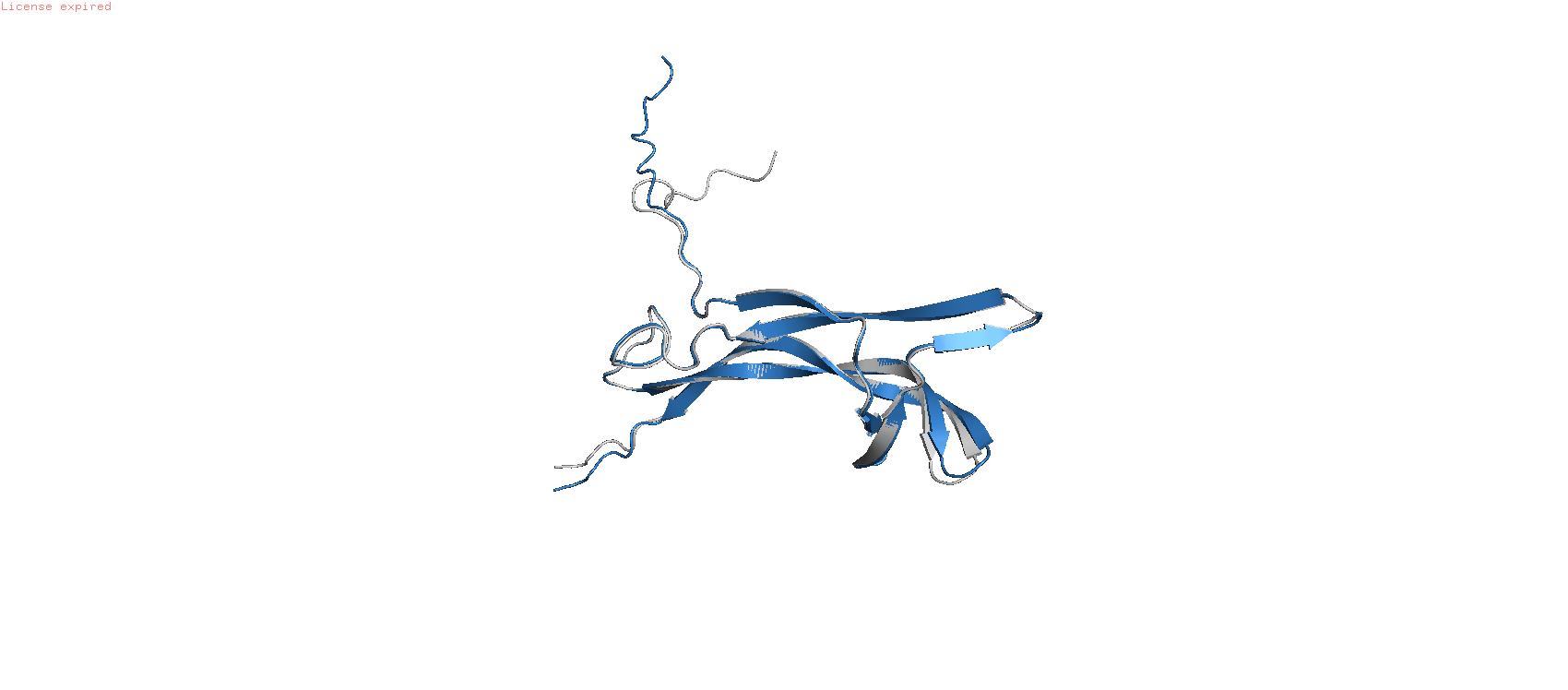


Superimposition of βNGF, βNGF (-)


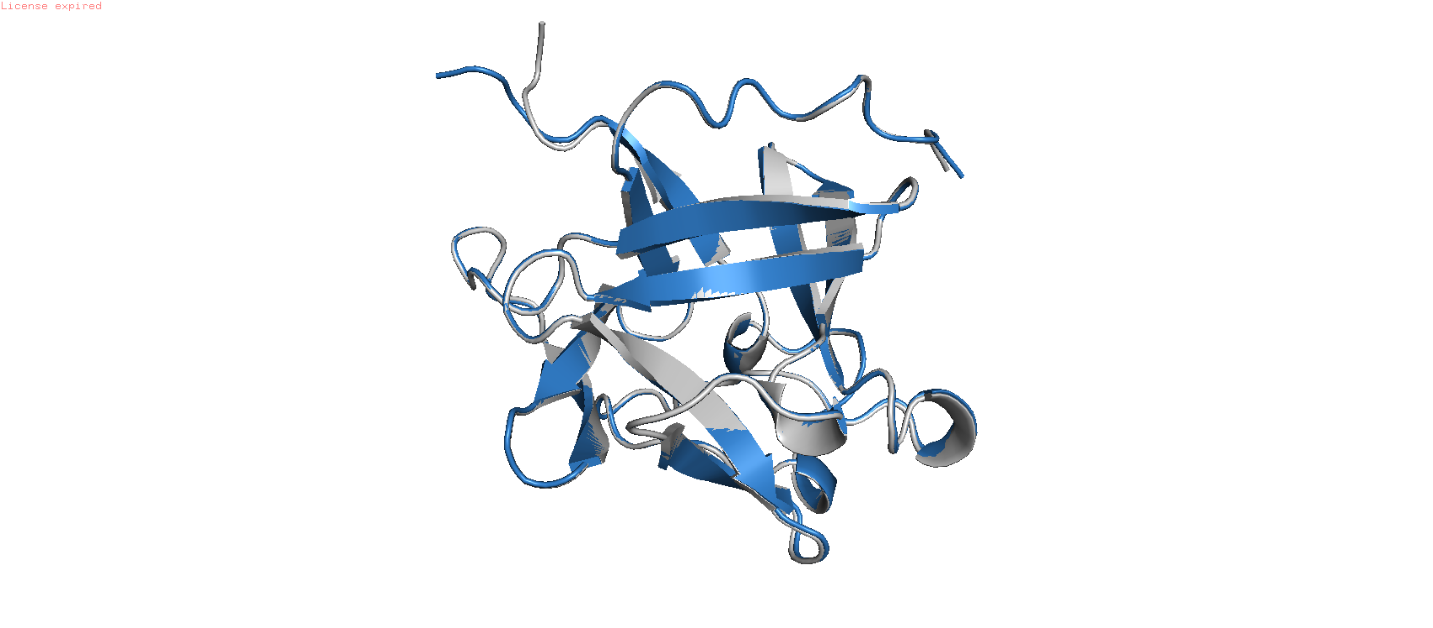


Superimposition of NTD, NTD ≤ 2


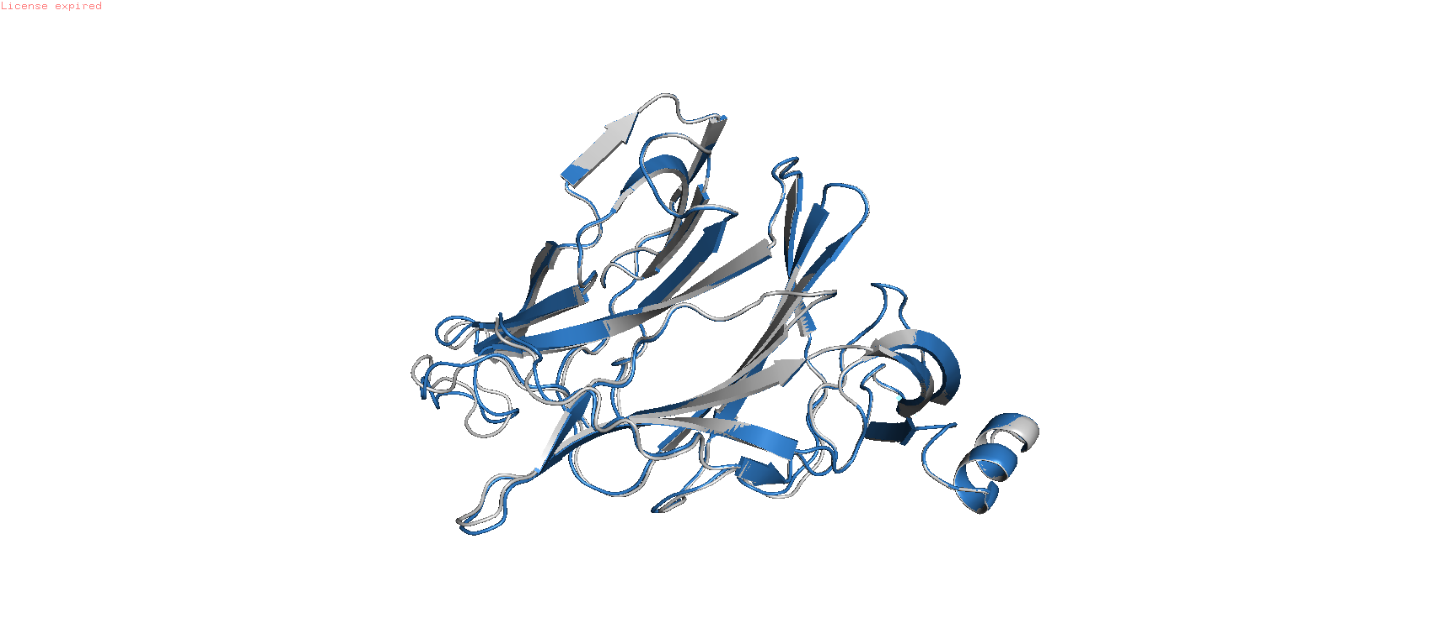


Superimposition of NTD, NTD ≤ 1


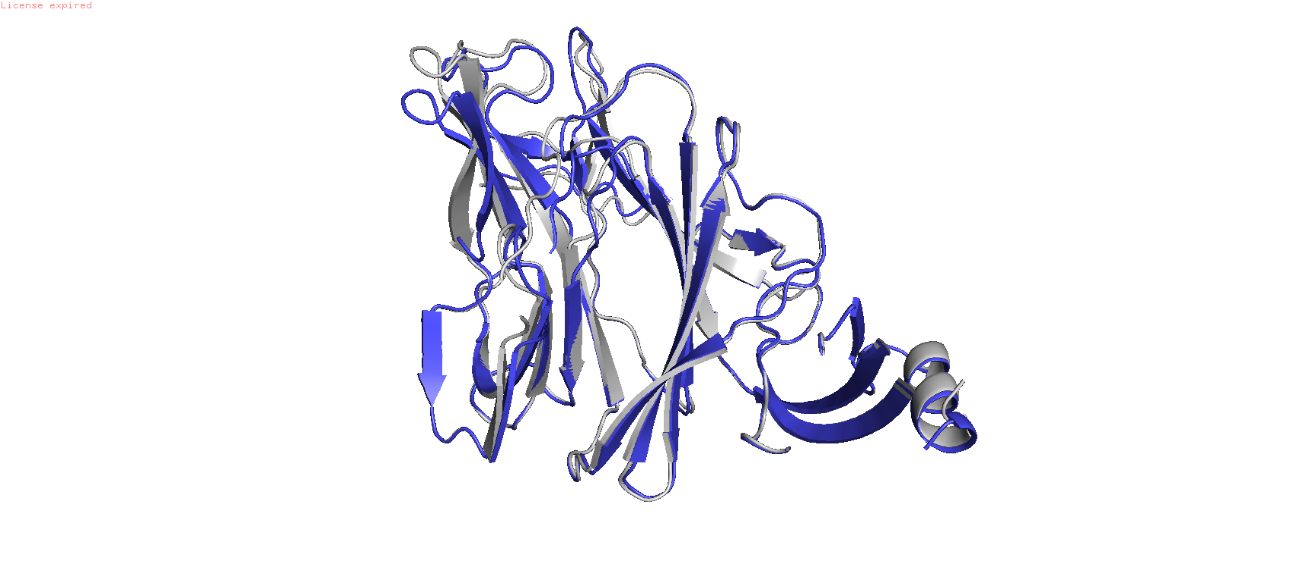


Superimposition of RBD, RBD ≤ 2


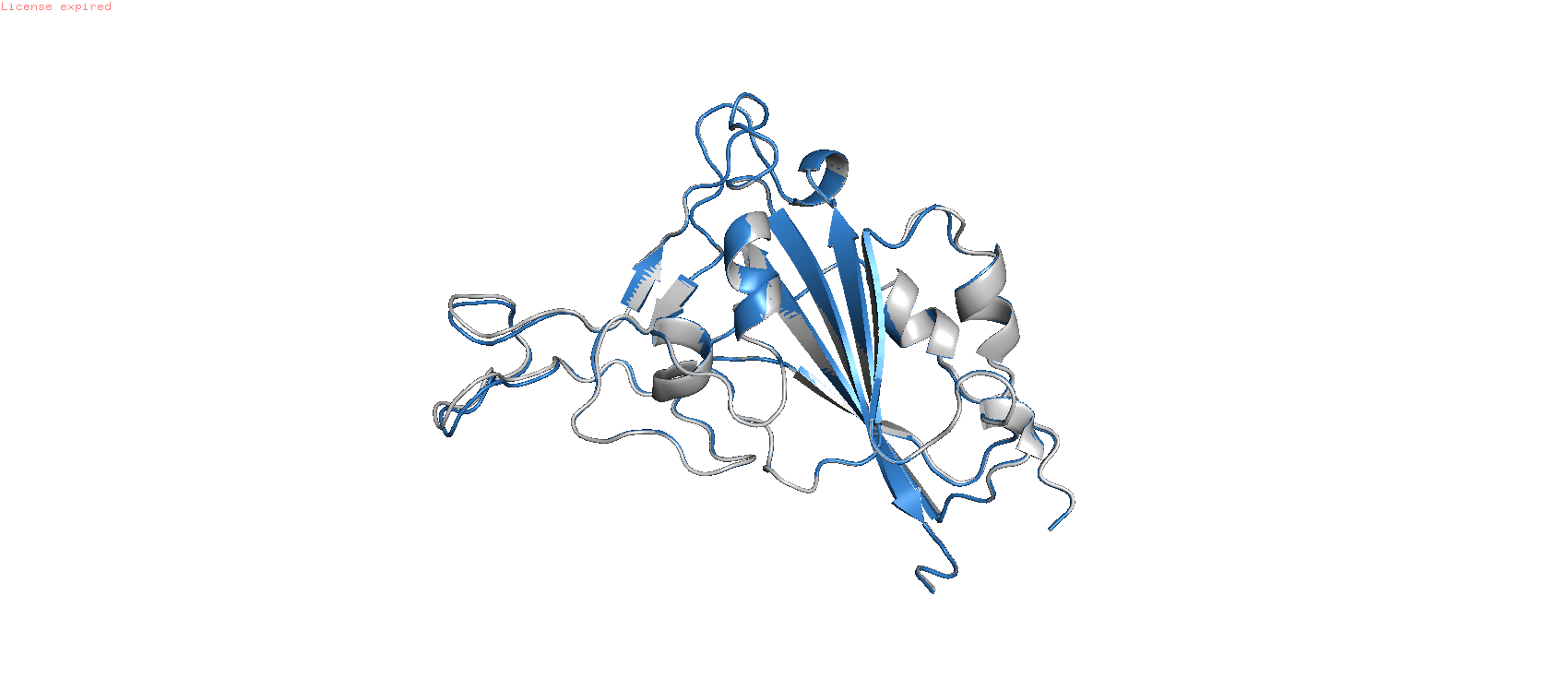


Superimposition of RBD, RBD ≤ 1


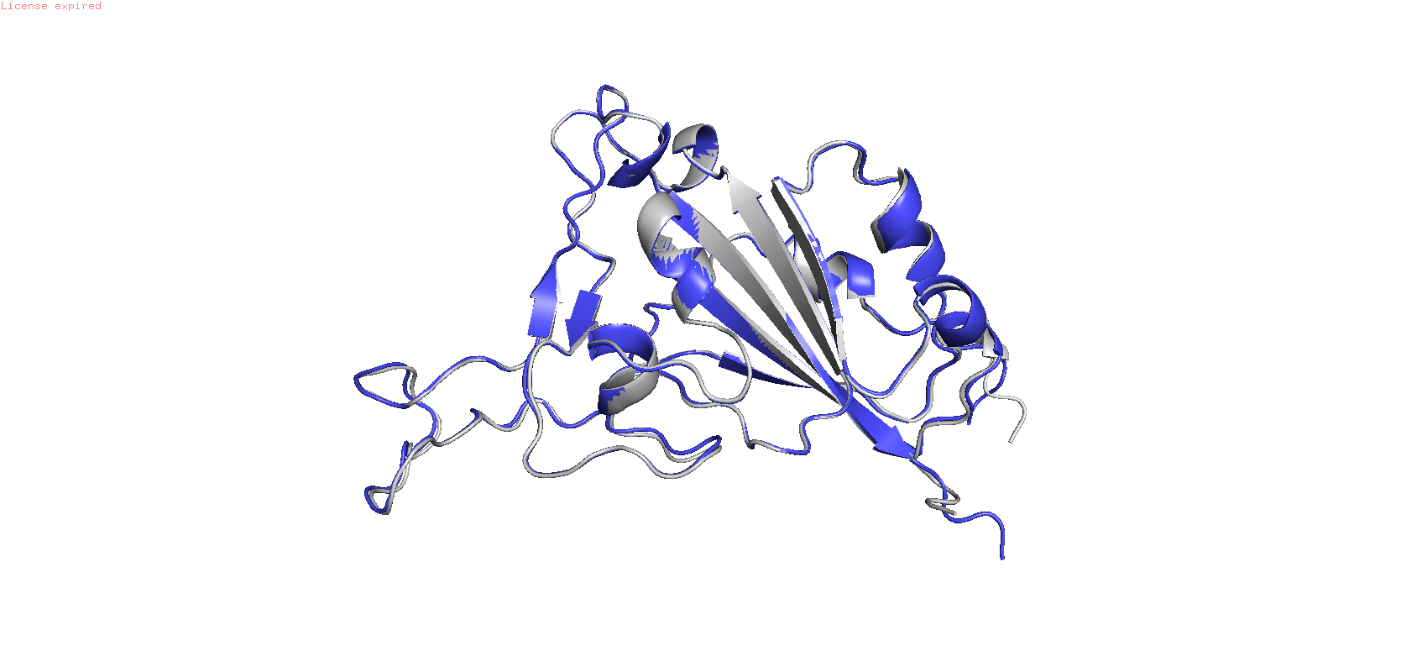


Superimposition of BoNT/A, BoNT/A ≤ 2


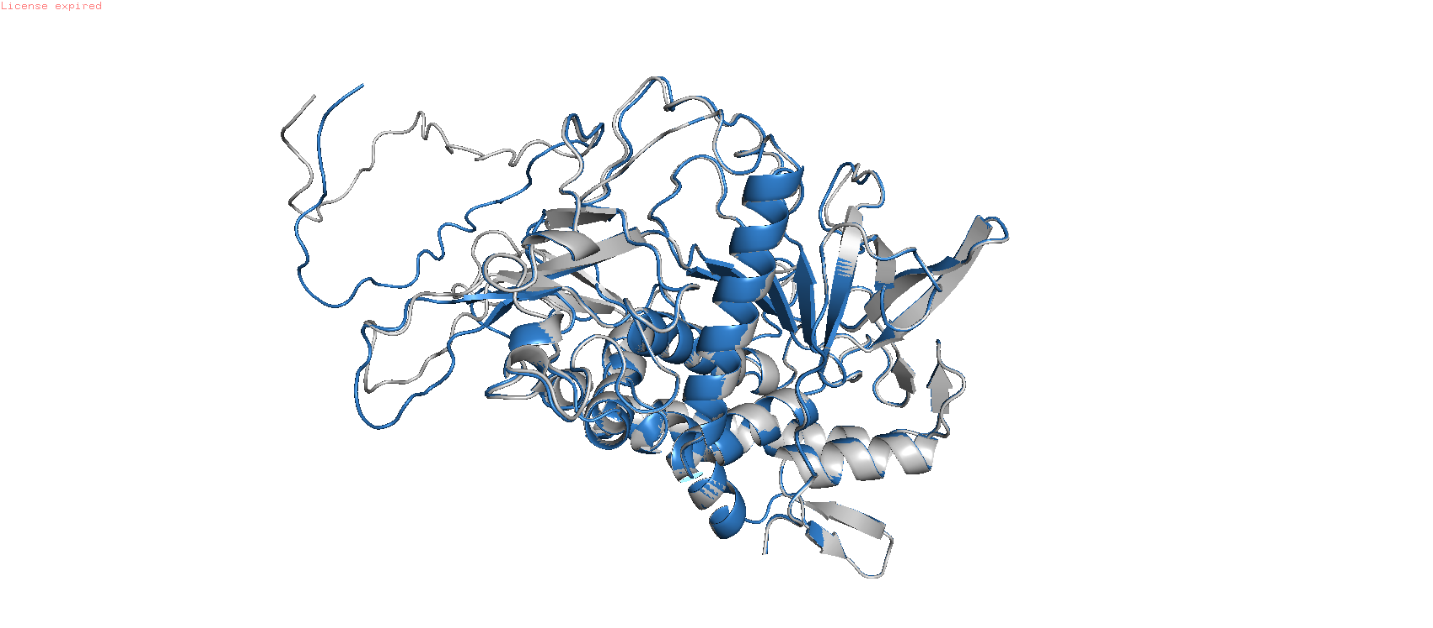


Superimposition of BoNT/A, BoNT/A ≤ 1


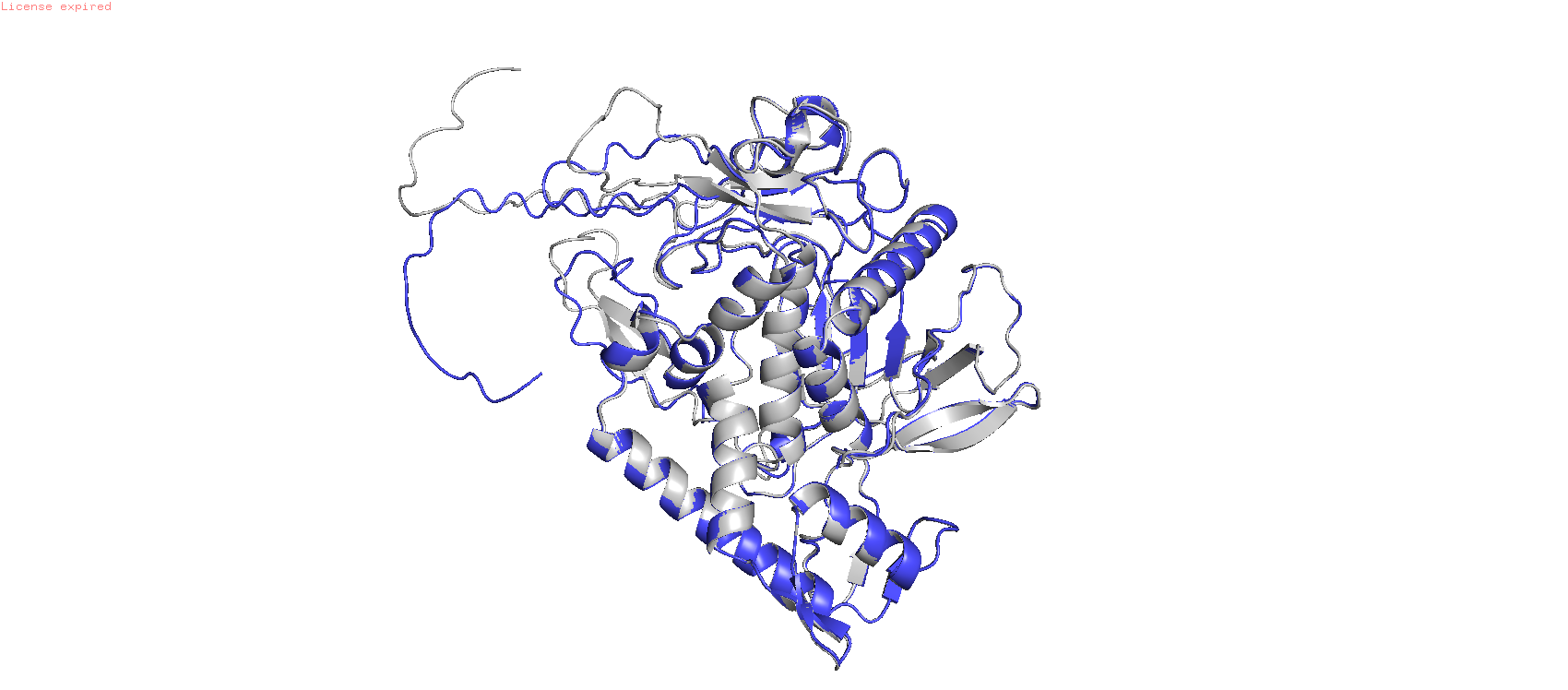

Supplement: Supplementary file 6 — Additional File 6: AlphaFold2-generated structures of wildtype and supercharged proteins. Superimposed AlphaFold2-generated protein structures of all proteins in the study. [file 12934_2024_2342_MOESM6_ESM.docx]
